# Methyl 2-{[(4-hydroxy­phen­yl)(methoxy­carbon­yl)meth­yl]amino­carbon­yl}ethano­ate hemihydrate

**DOI:** 10.1107/S1600536808005552

**Published:** 2008-03-05

**Authors:** M. Fazli Mohammat, Zurina Shaameri, A. Sazali Hamzah, N. Kamarulzaman, Hoong-Kun Fun, Suchada Chantrapromma

**Affiliations:** a Institute of Science, Universiti Teknologi MARA, 40450 Shah Alam, Selangor, Malaysia; b X-ray Crystallography Unit, School of Physics, Universiti Sains Malaysia, 11800 USM, Penang, Malaysia; c Department of Chemistry, Faculty of Science, Prince of Songkla University, Hat-Yai, Songkhla 90112, Thailand

## Abstract

In the structure of the title compound, C_13_H_15_NO_6_·0.5H_2_O, the water O atom lies on a twofold rotation axis. The methoxy­carbonyl­methyl and amino groups are essentially coplanar and the methoxy­carbonyl­methyl group makes a dihedral angle of 79.73 (10)° with the mean plane of the hydroxy­phenyl ring. The amino and methoxy­carbonyl­methyl groups are involved in an intra­molecular N—H⋯O hydrogen bond which generates an *S*(5) ring motif. In the crystal structure, mol­ecules are linked *via* N—H⋯O and O—H⋯O hydrogen bonds and weak C—H⋯O inter­actions into a two-dimensional network parallel to the (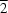
01) plane. The crystal structure is further stabilized by C—H⋯π inter­actions.

## Related literature

For bond-length data, see: Allen *et al.* (1987 ▶). For hydrogen-bond motifs, see: Bernstein *et al.* (1995 ▶). For details of the biological properties of compounds containing tetra­mic acid, see for example: Iida *et al.* (1986 ▶); Matkhalikova *et al.* (1969 ▶); Reddy & Rao (2006 ▶); Reiner (2007 ▶); Royles (1996 ▶). For the syntheses of compounds containing tetra­mic acid units, see for example: Steglich (1989 ▶); Royles (1996 ▶).
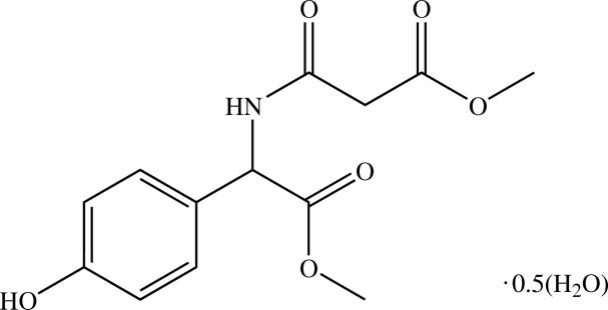


## Experimental

### 

#### Crystal data

C_13_H_15_NO_6_·0.5H_2_O
*M* *_r_* = 290.27
Monoclinic, 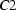

*a* = 22.7764 (12) Å
*b* = 5.3046 (3) Å
*c* = 13.0686 (6) Å
β = 117.612 (3)°
*V* = 1399.11 (13) Å^3^
*Z* = 4
Mo *K*α radiation
μ = 0.11 mm^−1^
*T* = 100.0 (1) K
0.41 × 0.19 × 0.04 mm

#### Data collection

Bruker SMART APEX2 CCD area-detector diffractometer
Absorption correction: multi-scan (*SADABS*; Bruker, 2005 ▶) *T*_min_ = 0.956, *T*_max_ = 0.996
9426 measured reflections
2248 independent reflections
1884 reflections with *I* > 2σ(*I*)
*R*_int_ = 0.035

#### Refinement

*R*[*F* ^2^ > 2σ(*F* ^2^)] = 0.040
*wR*(*F* ^2^) = 0.090
*S* = 1.06
2248 reflections
200 parameters
1 restraint
H atoms treated by a mixture of independent and constrained refinement
Δρ_max_ = 0.39 e Å^−3^
Δρ_min_ = −0.24 e Å^−3^

### 

Data collection: *APEX2* (Bruker, 2005 ▶); cell refinement: *APEX2*; data reduction: *SAINT* (Bruker, 2005 ▶); program(s) used to solve structure: *SHELXTL* (Sheldrick, 2008 ▶); program(s) used to refine structure: *SHELXTL*; molecular graphics: *SHELXTL*; software used to prepare material for publication: *SHELXTL* and *PLATON* (Spek, 2003 ▶).

## Supplementary Material

Crystal structure: contains datablocks global, I. DOI: 10.1107/S1600536808005552/sj2467sup1.cif

Structure factors: contains datablocks I. DOI: 10.1107/S1600536808005552/sj2467Isup2.hkl

Additional supplementary materials:  crystallographic information; 3D view; checkCIF report

## Figures and Tables

**Table 1:** Hydrogen-bond geometry (Å, °)

*D*—H⋯*A*	*D*—H	H⋯*A*	*D*⋯*A*	*D*—H⋯*A*
O1*W*—H1*W*⋯O1^i^	0.95 (4)	1.86 (3)	2.803 (3)	170 (3)
N1—H1*N*1⋯O1*W*	0.91 (3)	2.14 (3)	3.002 (3)	157 (2)
N1—H1*N*1⋯O3	0.91 (3)	2.28 (3)	2.669 (3)	105 (2)
O6—H1*O*6⋯O2^ii^	0.89 (4)	1.75 (3)	2.638 (2)	171 (3)
C2—H2*A*⋯O1*W*	0.97	2.49	3.363 (3)	150
C2—H2*B*⋯O6^ii^	0.97	2.34	3.146 (3)	140
C6—H6*B*⋯O6^iii^	0.96	2.49	3.420 (3)	162
C7—H7*B*⋯*Cg*1^iv^	0.96	2.68	3.574 (3)	155
C10—H10⋯*Cg*1^ii^	0.93	3.01	3.717 (2)	134

Symmetry codes: (i) 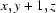; (ii) 
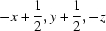
; (iii) 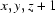; (iv) 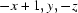. *Cg*1 is the centroid of the C8–C13 phenyl ring.

**Fig. 1.**
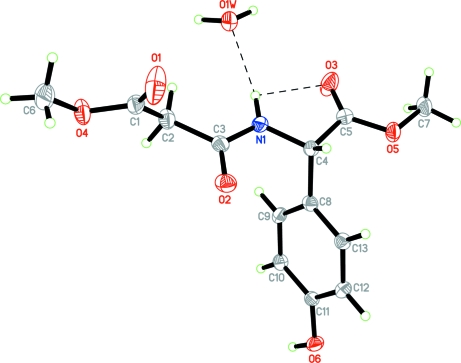
The molecular structure of (I), showing 50% probability displacement ellipsoids and the atomic numbering. N—H···O hydrogen bonds are drawn as dashed lines.

**Fig. 2.**
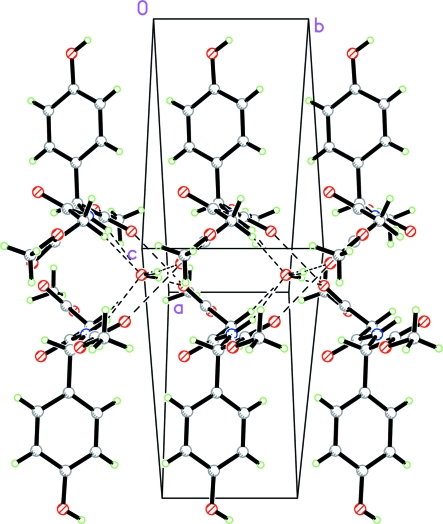
The crystal packing of (I), viewed along the [102] direction. Hydrogen bonds are drawn as dashed lines.

## supplementary crystallographic information

### Comment

Natural products containing tetramic acid goups continue to attract the interest of chemists and biologists due to their challenging structures and remarkable biological properties (Iida *et al.*, 1986; Matkhalikova *et al.*, 1969; Reddy & Rao, 2006; Reiner, 2007; Royles, 1996). Among these, tetramic acids carrying an aromatic substituent on the ring are rarely found in nature (Reddy & Rao, 2006). The title compound, C_13_H_15_NO_6_, can act as an essential intermediate in the synthesis of compounds responsible for the orange-yellow colour of plasmodia from *Leocarpus fragilis* (Steglich, 1989; Royles, 1995). We have synthesized the title compound and its structure is reported here.

The asymmetric unit of the title compound contains one molecule of C_13_H_15_NO_6_ and half an H_2_O molecule with the O1W atom lying on a twofold rotation axis, (Fig. 1). The methoxycarbonylmethyl [C4/C5/C7/O3/O5] and the C3/N1/C4 amino sections of the molecule are essentially coplanar with a dihedral angle of 3.12 (10)° between them. An intramolecular N1—H1N1···O3 hydrogen bond (Fig. 1) generates an S(5) ring motif (Bernstein *et al.*, 1995) and contributes to this planarity.

In the 3-oxopropanoate moiety [C1–C3/C6/O1/O2/O4], atoms C1, C2, C6, O1 and O4 lie on the same plane with C1 deviating by a maximum of -0.017 (2) Å. Similarly atoms C3, O2, C4, C5, N1 and O3 lie on the same plane with the maximum deviation -0.058 (2) Å for C4. The dihedral angle between these two planes is 70.29 (11) Å. The methoxycarbonylmethyl moiety makes a dihedral angle of 79.73 (10) Å with the hydroxyphenyl ring. The water molecule links with the C_13_H_15_NO_6_ molecule *via* an N1—H1N1···O1W hydrogen bond (Fig. 1). All bond lengths and angles show normal values (Allen *et al.*, 1987).

In the crystal packing (Fig. 2), the molecules are stacked down both the [010] and [102] directions forming a two dimensional network parallel to the (-2 0 1) plane *via* N—H···O, O—H···O hydrogen bonds and weak C—H···O interactions (Table 1). The crystal is further stablized by C—H···π interactions (Table 1); *Cg*_1_ is the centroid of the C8–C13 phenyl ring.

### Experimental

The title compound was synthesized *via* condensation between an equimolar amount of hydroxyphenylglycine methylester (10.0 g, 60 mmol) and methylmalonate potassium salt (9.4 g, 60 mmol) in acetonitrile/water (140:40 ml) at 273 K. The mixture was stirred for 2 h in the presence of dicyclohexylcarbodiimide, which acted as a catalyst and a peptide-coupling agent. The white precipitate formed during the reaction was filtered and washed thoroughly with dichloromethane. The filtrate and the dichloromethane were combined and evaporated. The resulting crude product was partitioned between water and dichloromethane, and the dichloromethane extract was dried over anhydrous magnesium sulfate and evaporated. Colorless needle-shaped single crystals suitable for *X*-ray structure determination were obtained by slow evaporation of dichloromethane/petroleum ether (5:1 *v*/*v*) solution after several days (10.93 g, 65%).

### Refinement

The amino, hydroxyl and water hydrogen atoms were located in a difference map and refined isotropically. Hydrogen atoms attached to the carbon atoms were constrained in a riding motion approximation with d(C—H) = 0.93 Å, *U*_iso_=1.2*U*_eq_(C) for aromatic, 0.98 Å, *U*_iso_ = 1.2*U*_eq_(C) for CH, 0.97 Å, *U*_iso_ = 1.2*U*_eq_(C) for CH_2_, 0.96 Å, *U*_iso_ = 1.5*U*_eq_(C) for CH_3_ atoms. A rotating group model was used for the methyl groups. In the absence of significant anomalous scattering effects, a total of 1388 Friedel pairs were merged before final refinement.

### Crystal data

**Table:**

C_13_H_15_NO_6_·0.5H_2_O	*F*_000_ = 612
*M**_r_* = 290.27	*D*_x_ = 1.378 Mg m^−^^3^
Monoclinic, *C*2	Mo *K*α radiation λ = 0.71073 Å
Hall symbol: C 2y	Cell parameters from 2248 reflections
*a* = 22.7764 (12) Å	θ = 1.8–30.0º
*b* = 5.3046 (3) Å	µ = 0.11 mm^−^^1^
*c* = 13.0686 (6) Å	*T* = 100.0 (1) K
β = 117.612 (3)º	Needle, colorless
*V* = 1399.11 (13) Å^3^	0.41 × 0.19 × 0.04 mm
*Z* = 4	

### Data collection

**Table:**

Bruker SMART APEX2 CCD area-detector diffractometer	2248 independent reflections
Radiation source: fine-focus sealed tube	1884 reflections with *I* > 2σ(*I*)
Monochromator: graphite	*R*_int_ = 0.035
Detector resolution: 8.33 pixels mm^-1^	θ_max_ = 30.0º
*T* = 100.0(1) K	θ_min_ = 1.8º
ω scans	*h* = −29→31
Absorption correction: multi-scan(SADABS; Bruker, 2005)	*k* = −7→7
*T*_min_ = 0.956, *T*_max_ = 0.996	*l* = −18→18
9426 measured reflections	

### Refinement

**Table:**

Refinement on *F*^2^	Secondary atom site location: difference Fourier map
Least-squares matrix: full	Hydrogen site location: inferred from neighbouring sites
*R*[*F*^2^ > 2σ(*F*^2^)] = 0.040	H atoms treated by a mixture of independent and constrained refinement
*wR*(*F*^2^) = 0.090	*w* = 1/[σ^2^(*F*_o_^2^) + (0.0406*P*)^2^ + 0.4523*P*] where *P* = (*F*_o_^2^ + 2*F*_c_^2^)/3
*S* = 1.06	(Δ/σ)_max_ < 0.001
2248 reflections	Δρ_max_ = 0.39 e Å^−^^3^
200 parameters	Δρ_min_ = −0.24 e Å^−^^3^
1 restraint	Extinction correction: none
Primary atom site location: structure-invariant direct methods	

### Special details

**Table:**

Experimental. The data was collected with the Oxford Cyrosystem Cobra low-temperature attachment.
Geometry. All e.s.d.'s (except the e.s.d. in the dihedral angle between two l.s. planes) are estimated using the full covariance matrix. The cell e.s.d.'s are taken into account individually in the estimation of e.s.d.'s in distances, angles and torsion angles; correlations between e.s.d.'s in cell parameters are only used when they are defined by crystal symmetry. An approximate (isotropic) treatment of cell e.s.d.'s is used for estimating e.s.d.'s involving l.s. planes.
Refinement. Refinement of *F*^2^ against ALL reflections. The weighted *R*-factor *wR* and goodness of fit *S* are based on *F*^2^, conventional *R*-factors *R* are based on *F*, with *F* set to zero for negative *F*^2^. The threshold expression of *F*^2^ > σ(*F*^2^) is used only for calculating *R*-factors(gt) *etc*. and is not relevant to the choice of reflections for refinement. *R*-factors based on *F*^2^ are statistically about twice as large as those based on *F*, and *R*- factors based on ALL data will be even larger.

### Fractional atomic coordinates and isotropic or equivalent isotropic displacement parameters (Å^2^)

**Table:**

	*x*	*y*	*z*	*U*_iso_*/*U*_eq_	
O1W	0.5000	0.8858 (5)	0.5000	0.0242 (5)	
H1W	0.4734 (14)	0.998 (7)	0.518 (2)	0.058 (9)*	
O1	0.41331 (9)	0.1694 (5)	0.55170 (15)	0.0512 (6)	
O2	0.36387 (7)	0.1785 (3)	0.28492 (11)	0.0255 (3)	
O3	0.51628 (9)	0.7925 (4)	0.27318 (16)	0.0435 (5)	
O4	0.32338 (7)	0.3629 (4)	0.53519 (12)	0.0311 (4)	
O5	0.52565 (7)	0.5121 (3)	0.15432 (12)	0.0250 (3)	
O6	0.23720 (7)	0.3718 (3)	−0.23284 (11)	0.0232 (3)	
H1O6	0.2058 (14)	0.488 (7)	−0.250 (2)	0.046 (8)*	
N1	0.43133 (8)	0.5081 (4)	0.31026 (13)	0.0196 (4)	
H1N1	0.4513 (13)	0.650 (6)	0.351 (2)	0.039 (7)*	
C1	0.37123 (10)	0.3244 (4)	0.50613 (16)	0.0231 (5)	
C2	0.36758 (10)	0.5050 (5)	0.41546 (17)	0.0239 (4)	
H2A	0.3963	0.6477	0.4522	0.029*	
H2B	0.3226	0.5676	0.3728	0.029*	
C3	0.38810 (9)	0.3812 (5)	0.33240 (15)	0.0201 (4)	
C4	0.44916 (9)	0.4168 (4)	0.22326 (14)	0.0178 (4)	
H4	0.4684	0.2479	0.2450	0.021*	
C5	0.50120 (10)	0.5952 (4)	0.22279 (16)	0.0213 (4)	
C6	0.32471 (12)	0.1992 (6)	0.62502 (18)	0.0382 (6)	
H6A	0.3150	0.0295	0.5966	0.057*	
H6B	0.2921	0.2546	0.6473	0.057*	
H6C	0.3678	0.2051	0.6907	0.057*	
C7	0.57232 (10)	0.6792 (5)	0.14299 (18)	0.0275 (5)	
H7A	0.5530	0.8438	0.1209	0.041*	
H7B	0.5831	0.6152	0.0850	0.041*	
H7C	0.6119	0.6897	0.2155	0.041*	
C8	0.39035 (9)	0.4061 (4)	0.10253 (14)	0.0173 (4)	
C9	0.34208 (9)	0.5916 (4)	0.06352 (15)	0.0185 (4)	
H9	0.3447	0.7234	0.1124	0.022*	
C10	0.28956 (9)	0.5828 (4)	−0.04846 (15)	0.0186 (4)	
H10	0.2571	0.7071	−0.0738	0.022*	
C11	0.28619 (9)	0.3874 (4)	−0.12164 (14)	0.0171 (4)	
C12	0.33391 (9)	0.1994 (4)	−0.08248 (15)	0.0202 (4)	
H12	0.3312	0.0669	−0.1311	0.024*	
C13	0.38579 (9)	0.2087 (4)	0.02927 (15)	0.0189 (4)	
H13	0.4177	0.0820	0.0552	0.023*	

### Atomic displacement parameters (Å^2^)

**Table:**

	*U*^11^	*U*^22^	*U*^33^	*U*^12^	*U*^13^	*U*^23^
O1W	0.0252 (10)	0.0249 (12)	0.0262 (9)	0.000	0.0151 (9)	0.000
O1	0.0576 (11)	0.0695 (15)	0.0432 (9)	0.0427 (12)	0.0373 (9)	0.0351 (11)
O2	0.0250 (7)	0.0272 (8)	0.0224 (6)	−0.0058 (7)	0.0094 (6)	0.0016 (7)
O3	0.0540 (11)	0.0432 (12)	0.0547 (11)	−0.0279 (9)	0.0432 (10)	−0.0289 (9)
O4	0.0244 (7)	0.0455 (11)	0.0303 (7)	0.0075 (8)	0.0185 (6)	0.0128 (8)
O5	0.0257 (7)	0.0254 (8)	0.0320 (7)	−0.0051 (7)	0.0204 (6)	−0.0065 (7)
O6	0.0202 (7)	0.0268 (9)	0.0176 (6)	0.0031 (7)	0.0046 (5)	−0.0047 (6)
N1	0.0196 (8)	0.0240 (9)	0.0163 (7)	−0.0034 (8)	0.0093 (6)	−0.0012 (7)
C1	0.0205 (10)	0.0301 (13)	0.0188 (8)	0.0034 (9)	0.0093 (7)	0.0033 (8)
C2	0.0220 (9)	0.0286 (11)	0.0240 (9)	0.0026 (9)	0.0132 (8)	0.0046 (9)
C3	0.0159 (8)	0.0270 (11)	0.0148 (7)	0.0007 (9)	0.0048 (7)	0.0048 (9)
C4	0.0174 (8)	0.0200 (10)	0.0166 (7)	−0.0016 (8)	0.0083 (7)	−0.0003 (8)
C5	0.0200 (9)	0.0237 (11)	0.0199 (8)	−0.0016 (9)	0.0091 (7)	−0.0025 (9)
C6	0.0391 (13)	0.0542 (17)	0.0285 (10)	−0.0056 (14)	0.0218 (10)	0.0085 (13)
C7	0.0255 (10)	0.0311 (12)	0.0337 (10)	−0.0055 (10)	0.0203 (9)	−0.0048 (10)
C8	0.0175 (8)	0.0181 (10)	0.0175 (7)	−0.0034 (8)	0.0091 (7)	0.0017 (8)
C9	0.0218 (9)	0.0178 (10)	0.0184 (8)	−0.0006 (8)	0.0115 (7)	−0.0022 (8)
C10	0.0191 (9)	0.0168 (10)	0.0208 (8)	0.0018 (8)	0.0099 (7)	0.0011 (8)
C11	0.0157 (8)	0.0187 (10)	0.0168 (7)	−0.0006 (8)	0.0074 (7)	0.0003 (8)
C12	0.0233 (10)	0.0183 (10)	0.0196 (8)	−0.0002 (9)	0.0105 (8)	−0.0030 (8)
C13	0.0182 (9)	0.0174 (10)	0.0211 (8)	0.0011 (8)	0.0092 (7)	0.0000 (8)

### Geometric parameters (Å, °)

**Table:**

O1W—H1W	0.95 (3)	C4—C8	1.526 (2)
O1—C1	1.192 (3)	C4—H4	0.9800
O2—C3	1.236 (3)	C6—H6A	0.9600
O3—C5	1.199 (3)	C6—H6B	0.9600
O4—C1	1.326 (2)	C6—H6C	0.9600
O4—C6	1.449 (3)	C7—H7A	0.9600
O5—C5	1.329 (3)	C7—H7B	0.9600
O5—C7	1.443 (3)	C7—H7C	0.9600
O6—C11	1.364 (2)	C8—C9	1.385 (3)
O6—H1O6	0.89 (3)	C8—C13	1.390 (3)
N1—C3	1.331 (3)	C9—C10	1.397 (2)
N1—C4	1.456 (2)	C9—H9	0.9300
N1—H1N1	0.91 (3)	C10—C11	1.388 (3)
C1—C2	1.496 (3)	C10—H10	0.9300
C2—C3	1.516 (3)	C11—C12	1.386 (3)
C2—H2A	0.9700	C12—C13	1.390 (2)
C2—H2B	0.9700	C12—H12	0.9300
C4—C5	1.519 (3)	C13—H13	0.9300
			
C1—O4—C6	115.36 (18)	H6A—C6—H6B	109.5
C5—O5—C7	115.06 (18)	O4—C6—H6C	109.5
C11—O6—H1O6	112.7 (18)	H6A—C6—H6C	109.5
C3—N1—C4	120.25 (18)	H6B—C6—H6C	109.5
C3—N1—H1N1	120.7 (17)	O5—C7—H7A	109.5
C4—N1—H1N1	119.0 (17)	O5—C7—H7B	109.5
O1—C1—O4	122.7 (2)	H7A—C7—H7B	109.5
O1—C1—C2	125.06 (19)	O5—C7—H7C	109.5
O4—C1—C2	112.13 (18)	H7A—C7—H7C	109.5
C1—C2—C3	111.5 (2)	H7B—C7—H7C	109.5
C1—C2—H2A	109.3	C9—C8—C13	119.26 (16)
C3—C2—H2A	109.3	C9—C8—C4	121.39 (18)
C1—C2—H2B	109.3	C13—C8—C4	119.34 (18)
C3—C2—H2B	109.3	C8—C9—C10	120.66 (18)
H2A—C2—H2B	108.0	C8—C9—H9	119.7
O2—C3—N1	122.28 (19)	C10—C9—H9	119.7
O2—C3—C2	121.48 (19)	C11—C10—C9	119.58 (18)
N1—C3—C2	116.2 (2)	C11—C10—H10	120.2
N1—C4—C5	107.25 (16)	C9—C10—H10	120.2
N1—C4—C8	113.02 (16)	O6—C11—C12	117.66 (18)
C5—C4—C8	109.38 (15)	O6—C11—C10	122.39 (18)
N1—C4—H4	109.0	C12—C11—C10	119.95 (16)
C5—C4—H4	109.0	C11—C12—C13	120.13 (19)
C8—C4—H4	109.0	C11—C12—H12	119.9
O3—C5—O5	123.8 (2)	C13—C12—H12	119.9
O3—C5—C4	124.54 (19)	C12—C13—C8	120.40 (19)
O5—C5—C4	111.55 (18)	C12—C13—H13	119.8
O4—C6—H6A	109.5	C8—C13—H13	119.8
O4—C6—H6B	109.5		
			
C6—O4—C1—O1	−1.3 (3)	C8—C4—C5—O5	−63.6 (2)
C6—O4—C1—C2	−178.40 (19)	N1—C4—C8—C9	38.6 (3)
O1—C1—C2—C3	38.6 (3)	C5—C4—C8—C9	−80.8 (2)
O4—C1—C2—C3	−144.42 (18)	N1—C4—C8—C13	−142.68 (19)
C4—N1—C3—O2	3.3 (3)	C5—C4—C8—C13	97.9 (2)
C4—N1—C3—C2	−174.03 (16)	C13—C8—C9—C10	−0.5 (3)
C1—C2—C3—O2	50.6 (2)	C4—C8—C9—C10	178.14 (18)
C1—C2—C3—N1	−132.06 (19)	C8—C9—C10—C11	−0.7 (3)
C3—N1—C4—C5	−177.48 (17)	C9—C10—C11—O6	−178.20 (18)
C3—N1—C4—C8	61.9 (2)	C9—C10—C11—C12	1.5 (3)
C7—O5—C5—O3	−0.6 (3)	O6—C11—C12—C13	178.59 (19)
C7—O5—C5—C4	176.08 (16)	C10—C11—C12—C13	−1.2 (3)
N1—C4—C5—O3	−10.0 (3)	C11—C12—C13—C8	−0.1 (3)
C8—C4—C5—O3	113.0 (2)	C9—C8—C13—C12	0.9 (3)
N1—C4—C5—O5	173.45 (16)	C4—C8—C13—C12	−177.78 (18)

### Hydrogen-bond geometry (Å, °)

**Table:**

*D*—H···*A*	*D*—H	H···*A*	*D*···*A*	*D*—H···*A*
O1W—H1W···O1^i^	0.95 (4)	1.86 (3)	2.803 (3)	170 (3)
N1—H1N1···O1W	0.91 (3)	2.14 (3)	3.002 (3)	157 (2)
N1—H1N1···O3	0.91 (3)	2.28 (3)	2.669 (3)	105 (2)
O6—H1O6···O2^ii^	0.89 (4)	1.75 (3)	2.638 (2)	171 (3)
C2—H2A···O1W	0.97	2.49	3.363 (3)	150
C2—H2B···O6^ii^	0.97	2.34	3.146 (3)	140
C6—H6B···O6^iii^	0.96	2.49	3.420 (3)	162
C7—H7B···Cg1^iv^	0.96	2.68	3.574 (3)	155
C10—H10···Cg1^ii^	0.93	3.01	3.717 (2)	134

Symmetry codes: (i) *x*, *y*+1, *z*;  (ii) −*x*+1/2, *y*+1/2, −*z*; (iii) *x*, *y*, *z*+1; (iv) −*x*+1, *y*, −*z*.
